# Methods of measuring presynaptic function with fluorescence probes

**DOI:** 10.1186/s42649-021-00051-0

**Published:** 2021-03-17

**Authors:** Yeseul Jang, Sung Rae Kim, Sung Hoon Lee

**Affiliations:** College of Pharmacy, Chung-Ang University, Seoul, 06974 Republic of Korea

**Keywords:** Synaptic vesicles, Exo- and endocytosis, Presynaptic terminal, Fluorescence probes

## Abstract

Synaptic vesicles, which are endogenous to neurotransmitters, are involved in exocytosis by active potentials and release neurotransmitters. Synaptic vesicles used in neurotransmitter release are reused via endocytosis to maintain a pool of synaptic vesicles. Synaptic vesicles show different types of exo- and endocytosis depending on animal species, type of nerve cell, and electrical activity. To accurately understand the dynamics of synaptic vesicles, direct observation of synaptic vesicles is required; however, it was difficult to observe synaptic vesicles of size 40–50 nm in living neurons. The exo-and endocytosis of synaptic vesicles was confirmed by labeling the vesicles with a fluorescent agent and measuring the changes in fluorescence intensity. To date, various methods of labeling synaptic vesicles have been proposed, and each method has its own characteristics, strength, and drawbacks. In this study, we introduce methods that can measure presynaptic activity and describe the characteristics of each technique.

## Introduction

Neurotransmitters are stored in synaptic vesicles (SVs). When nerve cells are activated by action potential, extracellular calcium enters the cells by electrical depolarization; the concentration of calcium increases at presynaptic terminals, and SVs fuse with presynaptic membranes by exocytosis. At this time, the neurotransmitters are released at the synaptic cleft, and the SVs that release the neurotransmitters are reused in the presynaptic membrane through the endocytosis process.

There are hundreds to thousands of synaptic vesicles at one presynaptic terminal, and the SVs are divided depending on their release probability according to their electrical strength, such as readily releasable pools (RRPs released immediately in response to a small action potential), recycling pools (SVs released in response to a stronger action potential that is more persistent than RRPs), and resting pools (not released in response to a strong action potential) (Denker and Rizzoli 2010). The number of SVs, pool ratio of SVs, and endocytosis rates are displayed differently depending on the species and type of synapse (Gan and Watanabe 2018).

SV exocytosis plays a significant role in synaptic communication because neurotransmitters are released into synapses by SV exocytosis. SV endocytosis is crucial for synaptic transmission by maintaining SV pools and lowers total synaptic membrane tension, which is increased by SV exocytosis. Therefore, the function of presynapses can be investigated by measuring SV exo- and endocytosis.

Synaptic vesicles are very small in size (40 to 50 nm) and have round shapes. SV exocytosis occurs within a few milliseconds in response to action potential, and endocytosis occurs in various ways for several tens of milliseconds to tens of seconds depending on the endocytic mode. Therefore, it is necessary to develop microscopy with spatial and temporal resolution to observe SV exo-and endocytosis in living cells. Because the current resolution of optical microscopes is several hundred nanometers, direct observation of SV exo- and endocytosis in living neurons has not been performed.

As it is difficult to directly observe SV exo-and endocytosis due to resolution limitations, SV exo- and endocytosis can be measured with specific markers for SV labeling. For markers that enhance the selectivity of SVs, a higher signal-to-noise ratio and photobleaching makes it easier to observe SV exo- and endocytosis. Various methods of labeling SVs have been reported and developed, such as a dye for labeling SVs, fusion protein that is specific to synaptic vesicles and fluorescence proteins, and new material for labeling SVs without photobleaching (Kavalali and Jorgensen 2014).

## Dyes

### Styryl dyes

**Fig. 1:**
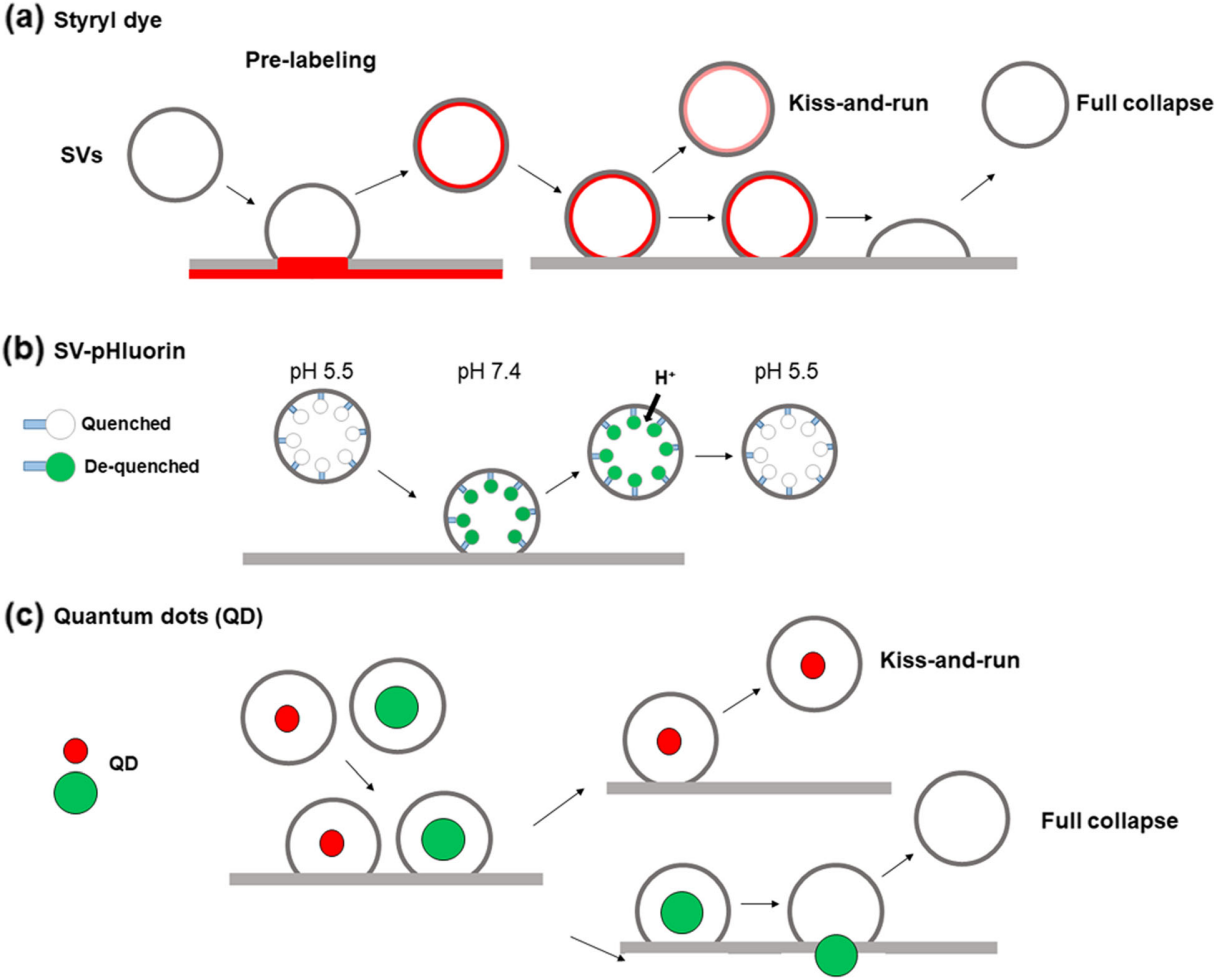
Optical measurement of presynaptic activity with fluorescent probes. Measurement of presynaptic activity with styryl dye (**a**), SV-pHluorin (**b**), and quantum dots (**c**)

Styryl dyes such as FM dyes are often used as amphipathic compounds for observing exo- and endocytosis in vesicles in various synapses (Betz et al. 1996). FM dyes are easily soluble in water, highly stable, bind to lipids, are retained by washouts, and do not penetrate cell membranes. Therefore, when synaptic vesicles are stained with FM dyes, they are not diffused out and are fused and retained in membranes only when exocytosis occurs in the SVs. SVs can be stained with FM dyes, and SV exo-and endocytosis can be measured with the fluorescence intensity changes. Endocytic modes are distinguished as kiss-and-run and full collapse by investigating detaining patterns (Fig. 1a) (Kavalali and Jorgensen 2014).

FM dyes are the easiest way to label SVs without genetic modifications or other additional methods, but can bind to nicotinic and muscarinic receptors and have non-selective effects, such as blocking mechanotransduction channels (Bewick and Betz 1994; Mazzone et al. 2006; Richards et al. 2000). The signal-to-noise ratio may not be as good as methods using other fluorescent probes. The interpretation of the results may differ based on methods such as excitation and emission wavelengths, and hydrophobicity of FM dyes is different depending on the FM dye types (Gaffield and Betz 2006; Hoopmann et al. 2012).

### CypHer5E (cypHer)

CypHer is a pH-dependent organic dye that exhibits excitation and emission wavelengths at 633 nm and 695 nm, respectively. CypHer quenches and dequenches in neutral pH and acidic conditions, respectively. It shows the opposite tendency to pHluorin, which is introduced in the following section. SV exocytosis reduces fluorescence and endocytosis increases fluorescence (Adie et al. 2003). SVs can be labeled with cypHer that is fused with SV proteins, such as vesicular GABA transporter (VGAT), synaptotagmin 1, or phospholipids (Hua et al. 2011; Kahms and Klingauf 2018; Martens et al. 2008). As cypHer is a dye, it is easy to use to label SVs and does not require genetic modification. When dye is added to a nerve cell culture medium and reacted for a certain period of time, SVs can be labeled and then SV exo-and endocytosis can be observed in living cells. However, due to the large size of cypHer particles, SVs can only be labeled when the pore size is larger than the cypHer particles during SV endocytosis. The photobleaching effect is observed in cypHer and needs to be corrected (Hua et al. 2011).

## pH-sensitive optical probes

SVs lumen maintains a weak acidity of pH 5.5. SV exocytosis causes synaptic vesicles to fuse with presynaptic membranes, increasing the pH to neutral. When SVs are taken up into presynaptic membranes via the SV endocytosis process, the lumen of SVs is restored to acidity by the activity of V-ATPase. pHluorin, as a pH-dependent fluorescent protein, exhibits a reversible characteristic in which fluorescence disappears when it is acidic and fluorescence is expressed when it is neutral. Exocytosis, which expresses pHluorin in the lumen of SVs, increases the SV lumen to neutrality. During endocytosis, pHluorin fluorescence disappears as the SV lumen is restored to acidic circumstances. Using these features, SV exo- and endocytosis can be measured in living cells (Fig. 1b) (Miesenbock et al. 1998).

pHluorin fused to various presynaptic proteins has been widely used to measure SV exo- and endocytosis, such as neurotransmitter release by fusion with specific SV proteins (Silm et al. 2019), observation of the kinetics of single SVs (Balaji and Ryan 2007; Leitz and Kavalali 2014), distinguishing SV exo- and endocytosis pools (Hua et al. 2011; Li et al. 2005), SV release probability (Morris 2006), and regulatory mechanism of SV exo- and endocytosis (Wu et al. 2016). pHluorin is further used in SV exocytosis and endocytosis in vivo, such as measuring presynaptic activity in the CNS and PNS by using animal models that express endogenous pHluorin via genetic recombination (Bozza et al. 2004; Wyatt and Balice-Gordon 2008) or synaptic activity in specific parts of the brain by adenovirus-containing pHluorin (Silm et al. 2019).

pHluorin is a fluorescent protein that shows green color. A pH-dependent fluorescent protein that shows other fluorescence wavelengths has also been developed (Martineau et al. 2017) using two different simultaneous imaging techniques to confirm synaptic function and pHluorin (Li and Tsien 2012; Raingo et al. 2012; Ramirez et al. 2012) and observe specific neurotransmitter dynamics (Silm et al. 2019).

pHluorin has the advantage of being able to observe the dynamics of SV proteins in living neurons (Kononenko and Haucke 2015; Villarreal et al. 2017). Using pH-dependent properties, pHluorin was also utilized in various studies to measure the pH of organelles such as lysosomes and the endoplasmic reticulum (Reifenrath and Boles 2018). pHluorin also measures autophagy flux as autophagosomes are fused to acidic lysosomes to form autophagolysosomes during the autophagy process (Oliva Trejo et al. 2020), and pHluorin measures lysosome acidity that is required for protease activity in living cells (Ponsford et al. 2020). pHluorin fused to optogenetic probes measures changes in pH in organelles in response to optogenetic acidification (Rost et al. 2015).

Although pHluorin is widely used in studies to measure the pH of organelles, it requires genetic modifications, and caution is required for interpreting SV endocytosis because a decline in pHluorin fluorescence intensity reflects both SV endocytosis and acidification rates of V-ATPase (Budzinski et al. 2011). Therefore, further research is necessary to distinguish SV endocytosis and acidification rates to accurately determine SV endocytosis, such as quenching experiments using acidic solutions (Wu et al. 2016). However, using pHluorin may not be appropriate to investigate trafficking of membrane proteins. α-Amino-3-hydroxy-5-methyl-4-isoxazolepropionic acid receptor (AMPAR) is a glutamatergic receptor that is expressed in postsynaptic membranes, and upon ligand binding, AMPAR internalizes and subsequently degrades due to the proteasome-mediated pathway (Widagdo et al. 2015). Therefore, tracking AMPAR internalization in living cells is important to study the physiological role of AMPAR. pHluorin fused to GluA2, which is a subunit of AMPAR, is utilized to track AMPAR internalization (Suresh and Dunaevsky 2017). However, it was suggested that the decline in pHluorin was caused by the effect of intracellular acidification rather than AMPAR trafficking (Rathje et al. 2013). Therefore, combining pHluorin fused with proteins and other methods may overcome the shortcoming of pHluorin to demonstrate the detailed mechanisms of membrane protein dynamics in living cells.

## Quantum dots (QDs)

QDs are nanometer-sized fluorescent materials that can label various colors depending on the size. QDs have a high signal-to-noise ratio and exhibit stable features without photobleaching (Alivisatos et al. 2005) for long-term target molecule tracking. In neurons, QDs are utilized to traffic synaptic proteins, such as glycine or AMPA receptors (Bats et al. 2007; Dahan et al. 2003).

In chromaffin cells, different fusion modes during the vesicle exocytosis process have been reported (Chiang et al. 2014), and fusion pores were measured with extra- or intracellular specific fluorescence probes and stimulated emission depletion (STED) microscopy imaging (Shin et al. 2018). As SV sizes (~ 50 nm) are smaller than vesicle sizes in chromaffin cells (~ 200 nm), it is not possible to measure pores during SV exo- and endocytosis. However, because QDs can adjust the size of particles, the pore size that is generated during SV exo- and endocytosis can be estimated. The pore size during SV exo- and endocytosis can be estimated by pre-labeling SVs with QDs. During SV exo- and endocytosis, QDs larger than the pore sizes are not be released and maintained inside of SVs, whereas QDs smaller than the pore size are released, causing loss of fluorescence signals (Zhang et al. 2009).

At presynaptic terminals, specific endocytic modes of SVs and dynamics of individual SVs were monitored with QDs (Fig. 1c) (Lee et al. 2012; Zhang et al. 2007), and simultaneous imaging of fluorescent probes that label SVs and QD emissions at 605 nm was used to monitor imaging of single SVs (Zhang 2013). In addition, real-time three-dimensional (3D) imaging with QD-labeled SVs exhibited the relationship between the SV position on the release probability and fusion mode (Park et al. 2012). Although QDs label SVs, detailed mechanisms of the affinity between QDs and SVs are not well understood, and an efficient method of labeling SVs with QDs has not yet been investigated (Zhang 2013).

## Calcium indicators

Depolarization activates voltage-gated calcium channels (VGCCs), and calcium subsequently enters presynaptic terminals. Calcium plays a crucial role in SV exo- and endocytosis at presynaptic terminals. At presynaptic terminals, calcium changes appear to occur at a higher rate and more rapidly than in some other neuronal compartments (Koester and Sakmann 2000), and the distribution of VGCCs determines SV exocytosis and presynaptic plasticity (Catterall and Few 2008; Dittman and Ryan 2019; Rebola et al. 2019). Unlike SV exocytosis, the role of calcium in SV endocytosis is controversial, such as contradictory results from species and synapse types (Hallermann 2014; Wu and Wu 2014). Among VGCCs, P/Q-type, N-type, and R-type calcium channels are expressed at presynaptic terminals (Dolphin and Lee 2020). Measuring calcium currents with patch clamps is an accurate method of determining calcium changes. However, only large-size nerve terminals, such as the calyx of Held and mossy fiber, are available to measure calcium currents. Therefore, fluorescence probes for labeling calcium are used to measure calcium dynamics at presynaptic terminals.

### Dyes

Presynaptic calcium variability was measured using fluorescent dyes such as Fura-2, Fluo-5F, and Oregon Green BAPTA (Tsien 1981; Tsien et al. 1985). However, due to low spatial resolution and distorted signals, these dye methods make it difficult to accurately measure calcium dynamics at presynaptic terminals. Therefore, they may be used in combination with other markers that can distinguish axon terminals (Kirischuk et al. 1999) or measure presynaptic calcium changes with dextran-conjugated dyes (Beierlein et al. 2004; Kreitzer et al. 2000).

### Genetically encoded calcium indicator (GECI)

GECI is one of the most widely used calcium measurement markers as a synthetic indicator for measuring calcium. GECI was initially utilized in a limited manner due to a low signal-to-noise ratio and slow response, but now is widely used with many improvements (Grienberger and Konnerth 2012). Calcium binding to GECI causes conformational rearrangement and fluorescence (Perez Koldenkova and Nagai 2013), and GECI makes it easy to observe calcium for a few days in vitro or in vivo by genetic modifications (genetically encoded indicators of neuronal activity). Camgaroo (Baird et al. 1999), pericam (Nagai et al. 2001), and GCaMP (Nakai et al. 2001) were developed from GECI as single-fluorescent protein calcium sensors, and GCaMP is widely used for experiments (Tian et al. 2009).

GCaMP can be used to measure calcium changes in sub-organelles, and especially GCaMP fused to presynaptic proteins measures presynaptic calcium changes in vitro and in vivo (Dreosti et al. 2009). In addition, expressing GCaMP in specific neurons in vivo revealed molecular mechanisms involved in the regulation of neurotransmitter release (Sgobio et al. 2014), and the relationship between SV release and presynaptic calcium changes was clarified by simultaneous imaging of two different fluorescence probes for GCaMP and red-shifted reporter of SVs (Jackson and Burrone 2016; Li et al. 2011). GCaMP was developed to more accurately measure presynaptic calcium changes in living neurons (Brockhaus et al. 2019). However, animals expressing GCaMP displayed aberrant current activity and suppression of SV probability (Singh et al. 2018; Steinmetz et al. 2017), suggesting that artifacts derived from GCaMP overexpression in vivo should be assessed.

**Fig. 2:**
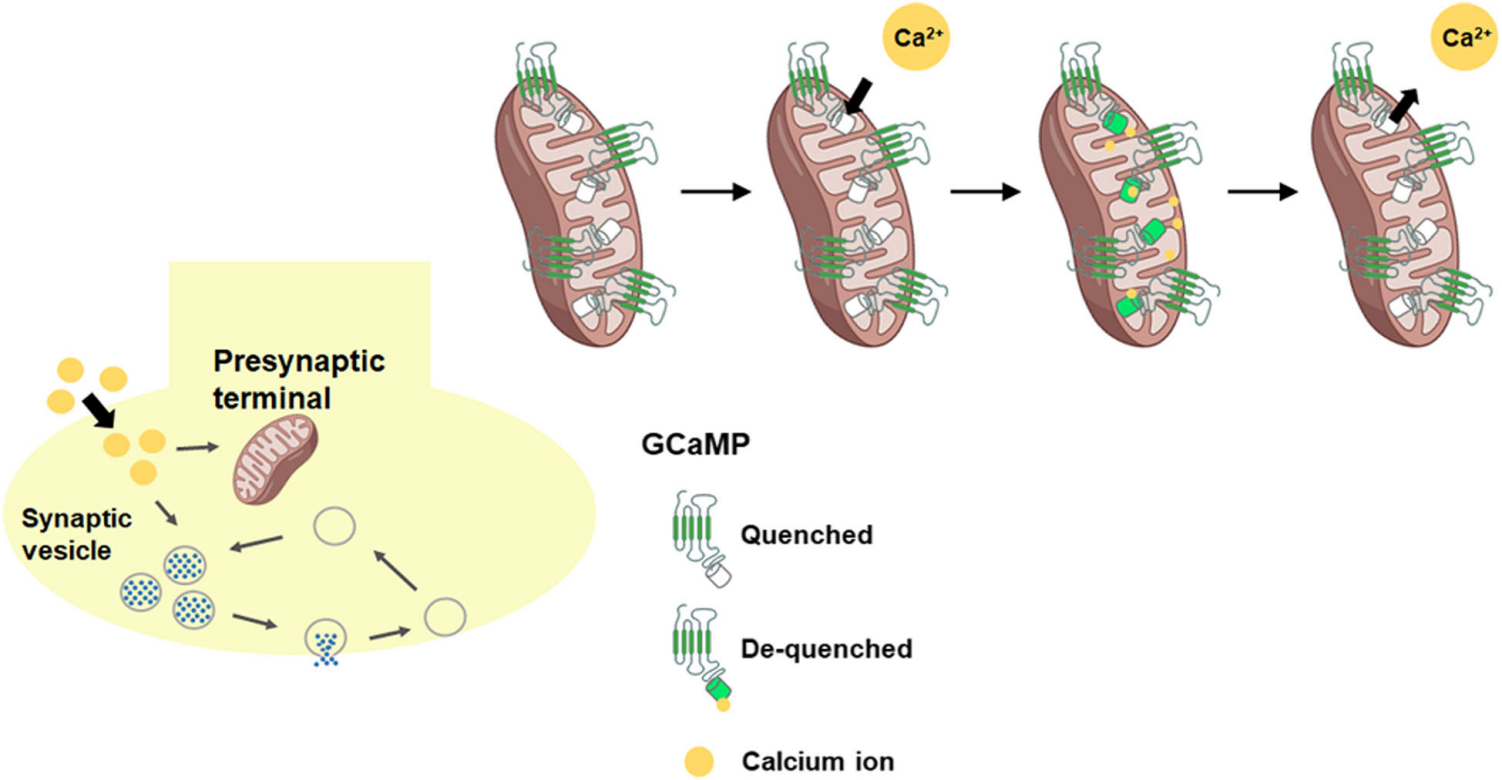
Measuring mitochondrial calcium with GCaMP

In recent studies, mitochondria have demonstrated a regulatory role in presynaptic transmission by buffering calcium levels at presynaptic terminals (Fig. 2) (Kwon et al. 2016), and mitochondrial calcium is involved in several neurodegenerative diseases (Jung et al. 2020). Exploring the links between mitochondrial calcium channels or exchangers and presynaptic activity will be an interesting area of future research.

## Conclusion

At presynaptic terminals, SV exocytosis is required to release neurotransmitters. SVs are locally recycled to maintain SV pools to continuously release neurotransmitters in response to repetitive action potentials. As SV exo- and endocytosis occurs repetitively at presynaptic terminals, exchanges between intracellular and extracellular substances were more active than in other neuronal compartments. Therefore, SV exo- and endocytosis is crucial for presynaptic activity. Method for measuring presynaptic activity has pros and cons. The method using dye has the advantage that it labels SVs by simply incubation without genetic modification; however, the signal to noise ratio is low. pHluorin measures SV protein dynamics at physiological condition in living neurons, but it requires genetic manipulation and decline of pHluorin intensity reflects SVs endocytosis and acidification rate. QDs have a high signal-to-noise ratio and stable features without photo-bleaching, enable to track SVs in long time. However, QD-induced biological or environmental toxicity has been concerned. Calcium indicator has been widely utilized for measuring presynaptic activity, but calcium channel activity may not accurately reflect SV release (Sames et al. 2013). Dynamics of SV exo- and endocytosis were variously observed according to the species, stimulus intensity, and presynapse type. In addition, because SV sizes are too small to directly observe, fluorescence probes for labeling SVs may affect SV dynamics results.

## Future direction

Fluorescence probes that selectively label SVs with high signal-to-noise ratio but without photobleaching are required to accurately understand the dynamics of SVs. In addition, measuring the neurotransmitter release in living neurons or brains would be the most accurate way to measure presynaptic activity. Several fluorescence probes or optical sensors have been developed, such as glutamate optical sensors (Gubernator et al. 2009) and fluorescent false neurotransmitters (Namiki et al. 2007). These probes enable measuring neurotransmitter release by visualization of exocytosis.

In addition, the development of a fluorescence probe that can label neurotransmitter release in neuronal compartment or brain tissues enables precise measurement of presynaptic activity. Furthermore, developing microscopy with higher spatial and temporal resolutions is necessary to measure SV dynamics. Using new probes to label SVs and ultra-high resolution fluorescence microscopy, new models of SV pools and SV dynamics have been suggested. A thorough understanding of the dynamics of SVs may facilitate the development of therapeutic strategies to treat neuropsychiatric or neurological diseases that are derived from abnormal presynaptic activity.

## Data Availability

The datasets used and/or analysed during the current study are available from the corresponding author on reasonable request.
